# In *Medicago truncatula*, water deficit modulates the transcript accumulation of components of small RNA pathways

**DOI:** 10.1186/1471-2229-11-79

**Published:** 2011-05-10

**Authors:** Cláudio Capitão, Jorge AP Paiva, Dulce M Santos, Pedro Fevereiro

**Affiliations:** 1 Laboratório de Biotecnologia de Células Vegetais, Instituto de Tecnologia Química e Biológica, Universidade Nova de Lisboa, Apartado 127, 2781-901 Oeiras, Portugal; 2 Instituto de Investigação Científica e Tropical, Centro das Florestas e Produtos Florestais, Tapada da Ajuda, 1349-017 Lisboa, Portugal; 3 Instituto de Investigação Científica e Tropical, Centro de Veterinária e Zootecnia, Av. Universidade Técnica 1300-477 Lisboa, Portugal; 4 Departamento de Biologia Vegetal, Faculdade de Ciências, Universidade de Lisboa, Campo Grande, 1749-016 Lisboa, Portugal

## Abstract

**Background:**

Small RNAs (sRNAs) are 20-24 nucleotide (nt) RNAs and are involved in plant development and response to abiotic stresses. Plants have several sRNA pathways implicated in the transcriptional and post-transcriptional silencing of gene expression. Two key enzyme families common to all pathways are the Dicer-like (DCL) proteins involved in sRNAs maturation and the Argonautes (AGOs) involved in the targeting and functional action of sRNAs. Post-transcriptional silencing mediated by AGOs may occur by cleavage or translational repression of target mRNA's, while transcriptional silencing may be controlled by DNA methylation and chromatin remodeling. Thus far, these gene families have not been characterized in legumes, nor has their involvement in adaptation to water deficit been studied.

**Results:**

A bioinformatic search in *Medicago truncatula *genome databases, using *Arabidopsis thaliana *AGO and DCL cDNA and protein sequences, identified three sequences encoding for putative Dicer-like genes and twelve sequences encoding for putative Argonaute genes. Under water deficit conditions and mainly in roots, MtDCL1 and MtAGO1, two enzymes probably involved in the processing and activation of microRNAs (miRNAs), increased their transcript levels. mir162 which target DCL1 mRNA and mir168 which target AGO1 mRNA reduced their expression in the roots of plants subjected to water deficit. Three putative genes, MtDCL3, MtAGO4b and MtAGO4c probably involved in DNA methylation mechanisms, increased their mRNA levels. However, the mRNA levels of MtAGO6 reduced, which probably encodes a protein with functions similar to MtAGO4. MtAGO7 mRNA levels increased and possibly encodes a protein involved in the production of trans-acting small interfering RNAs. The transcript abundance of MtAGO12a, MtAGO12b and MtAGO12c reduced under water deprivation. Plants recovered from water deprivation reacquire the mRNA levels of the controls.

**Conclusions:**

Our work demonstrates that in *M. truncatula *the transcript accumulation of the components of small RNA pathways is being modulated under water deficit. This shows that the transcriptional and post-transcriptional control of gene expression mediated by sRNAs is probably involved in plant adaptation to abiotic environmental changes. In the future this will allow the manipulation of these pathways providing a more efficient response of legumes towards water shortage.

## Background

In plants, the transcriptional and post-transcriptional regulation of gene expression mediated by sRNAs [1] is involved in several biological processes, ranging from organ differentiation to biotic and abiotic stress responses [2-4]. Small RNAs are divided into two main classes based on their biogenesis: the small interfering RNAs (siRNAs) are processed from perfect and long double-stranded RNAs while miRNAs are processed from single-stranded RNA transcripts that fold back onto themselves producing an imperfectly double-stranded stem loop [5]. The endogenous siRNAs are divided into trans-acting-siRNAs (ta-siRNAs) and heterochromatic siRNAs (hc-siRNAs) [6].

The pathways of gene silencing mediated by sRNAs share, in plants, four consensus biochemical steps [7]: (1) the biosynthesis of a double strand RNA (dsRNA); (2) the cutting of the dsRNA by a Dicer-like protein (DCL) in 18-25 nt-long sRNAs; (3) the O-methylation of the sRNAs by Hua Enhancer (HEN1), to protect them from degradation through the Small RNA Degrading Nuclease (SDN) class of exonucleases [8]; and (4) the integration of the sRNAs into an Argonaute (AGO) that associates with other proteins to promote gene silencing by partially or fully complementation with target RNA or DNA.

Plants have at least four different DCL proteins and each generates predominantly a particular class of sRNAs: DCL1 cleaves the imperfect double-stranded stem loop generating the miRNAs with around 21-nt [9]; DCL2 produces viral siRNAs 22-nt long [10]; DCL3 generates hc-siRNAs with 24-nt [10]; and DCL4 generates ta-siRNAs 21-nt long [11].

Plant DCLs contain six domains: one PAZ, two RNaseIII, one DEAD-helicase box (DEXD/H-box), one DUF283, at least one double-stranded RNA-binding (dsRB) domain and one Helicase-C domain [12]. The PAZ domain binds to double-stranded RNAs at the 3' end [13]. The two RNaseIII domains form an intramolecular dimer and the active site of each domain cleaves the dsRNA [14]. The DExD/H-box domain might have an auto-inhibitory function, because removal of this domain increases the cleavage rate of the human dicer [14]. The DUF283 domain displays affinity to bind the double-stranded RNA-binding domains of the *A. thaliana *dsRNA binding proteins (DRBs) [15] suggesting a functional role in the selection of the small RNA processing pathway.

The *A. thaliana *and *Oryza sativa *genomes have been completely sequenced and annotated [16,17]. These plant species encode ten and eighteen AGOs, respectively [16,17]. Both species share common phylogenetic related AGOs that are divided in three clades [18]. In *A. thaliana *some AGOs are well studied, for example AGO1 binds the miRNAs to mediate the cleavage of targets mRNAs and together with AGO10 both promote the translational repression of the targets but with different selectivity for the miRNAs [19,20]. AGO4, AGO6 and AGO9 fall in another clade and they are associated with hc-siRNAs to control DNA methylation [21]. AGO7 in the last clade is implicated in the production of the ta-siRNAs [22].

The AGO proteins generally contain one variable N-terminal region and one conserved C-terminal region constituted by the PAZ, middle (MID) and PIWI domains [23]. The PAZ domain binds to the 3' end of the guide strand of the sRNAs. The PIWI domain is responsible for the Argonaute slicer activity. The cleavage activity is carried out by the active site on the PIWI domain usually presenting an Asp-Asp-His (DDH) motif [19,24]. The slicer activity of Argonaute requires a perfect complementarity around the cleavage site of the guide-target duplex [25]. The 5' phosphate group of the sRNA guide strand is buried in a deep pocket at interface between the MID domain and PIWI domain [23].

In *A. thaliana*, the sRNAs association with the Argonaute proteins is based on the recognition of the 5' end nucleotide. This specificity is mediated by the MID domain [26]. For example AGO1 binds mainly to RNAs with a uridine at their 5' end, whereas AGO2, AGO4, AGO6 and AGO9 recruit RNAs with a 5' end adenosine and the AGO5 predominantly binds to sRNAs with a cytosine [21,26].

The biogenesis of miRNAs is under feedback regulation such that two key players are themselves regulated by miRNAs. DCL1 mRNA has a complementary sequence for miR162, which leads to the cleavage of DCL1 mRNA [27]. Likewise, AGO1 mRNA contains a complementary sequence for miR168 which leads to AGO1-mediated cleavage of AGO1 mRNA [28].

*Medicago truncatula *is a model legume [29], and its genome is almost completely sequenced (accessed 2 April 2010) [30]. However, almost nothing is known about the identification and function of AGO and DCL genes in legumes species. In *M. truncatula *several sRNAs were found to be differentially expressed in different organs and abiotic stress conditions [2,3,31,32]. Recently we described the up-regulation of miR398a/b and miR408 under water deficit and the corresponding down regulation of their respective targets, COX5b and plantacyanin [4]. However, no studies have been reported implicating the modulation of small RNA pathways in response to either water deficit or any other abiotic stress in legumes.

In the present study we identify three putative DCL and twelve putative AGO genes in the *M. truncatula *genome. We also established their phylogenetic relationship with the *A. thaliana *DCLs (AtDCLs) and AGOs (AtAGOs) and performed their domain characterization. The mRNA levels of these genes were quantified by quantitative real time PCR (qPCR) in vegetative growing plants under water deficit conditions. Our results show that the mRNA levels of the identified AGO and DCL genes are modulated when *M. truncatula *is subjected to water deprivation.

## Methods

### Plant material, growth and treatment conditions

*Medicago truncatula *Gaertn. cv. Jemalong seeds were scarified and sterilized in concentrated anhydrous sulfuric acid for 15 minutes according to Araújo et al [33]. After thoroughly washing with sterile water, seeds were placed on soaked filter paper in Petri dishes in the dark at 24°C. Three days later the seeds were transferred to a growth chamber (thermoperiod of 25/18°C, photoperiod of 16/8 h day/night, relative humidity of 40% and a Photosynthetic Photon Flux Density (PPFD) of 500μmol m^−2 ^s^−1^). One week old seedlings were transferred to vermiculite for 2 weeks and then individually transferred to 0.5 L pots with standard commercial non-sterile soil ("terra de Montemor", Horto do Campo Grande, Lisboa, Portugal). No nutrients were added to avoid any interference with the nodulation. The water status and physiological conditions of the different experimental groups were described in Nunes et al (2008) [34]. Briefly, eight-weeks-old plants were divided into four groups. The Control group (Ct, with a relative water content (RWC) = 80%) was constituted by plants maintained fully irrigated (maximum soil water capacity) (Additional file 1). The second and third groups were constituted by plants subjected to water deprivation for five (Moderate Water Deficit, MWD, RWC = 50%) and eight days (Severe Water Deficit, SWD, RWC = 30%). This severe time point was selected because above this point plants were unable to recover and quickly died. The fourth group consisted of the SWD plants that were re-watered for three days following water deficit, and so regained their original water status (Rec, RWC = 80%). All plants were nodulated when water uphold was started. The control plants always showed healthy nodules. At the severe water deficit condition most of the nodules senesced, but after 3 days of re-watering the nodules restart to develop.

### Identification of putative Dicer-like and Argonaute genes in *M. truncatula*

The mRNA and protein sequences of *A. thaliana *DCL and AGO genes were downloaded from the National Center for Biotechnology Information (NCBI) database [35] (Additional file 2). The algorithms BLASTn and tBLASTn were used to search the nucleotide sequence of the genes of interest, using a cut-off E-value of e^-20^, in the NCBI database [36], in the DFCI *M. truncatula *Gene index version 9.0 (MtGI9.0) and in the *M. truncatula *genome release version 3.0 (Mt3.0), using CViT-Blast and IMGAG-Blast [37].

### Characterization of the *M. truncatula *Dicer-like and Argonaute genes

The protein and nucleotide sequences of *M. truncatula *DCLs and AGOs were downloaded from MTGI9.0 and Mt3.0 databases. For MtAGO11 and MtAGO12b the annotation given by the Fgenesh algorithm was chosen. Fgenesh (Medicago matrix) is one of the gene prediction algorithms used in *M. truncatula *genome annotation by IMGAG (International Medicago Genome Annotation Group) [38,39]. In cases where the protein sequence was not available, the translation of the nucleotide sequence was done with the Translate software from Expert Protein Analysis System (ExPASy) [40]. The end of protein translation was considered when the first stop codon appeared. The longest amino acid sequence from the 6 possible reading frames was selected. The newly identified genes in this study were named based on the nomenclature used in *A. thaliana *and on their family phylogenetic relationships. Protein isoelectric point (Pi) was determined with the Protein Isoelectric Point software and calculation of protein molecular weight (MW) was performed using the Protein Molecular Weight software, both software are from the Sequence Manipulation Suite (SMS) package (version 2.0) [41,42].

### Protein domain search

Domain search was performed in the NCBI Conserved Domain Database (NCBI-CDD) [43-45]. The catalytic amino acids characteristic of the AGO proteins were identified aligning the PIWI domain sequences of *M. truncatula *and the known amino acid positions of *A. thaliana *AGO1 protein. The identification of the amino acid that separates the MID domain from the PIWI domain of MtAGOs protein sequences was obtained from the alignment of *Thermus thermophilus *AGO (gi:46255097)(PIWI start - 544), *Pyrococcus furiosus *AGO (gi:18976909)(PIWI start - 544), *Aquifex aeolicus *AGO (gi:15606619) (PIWI start - 487), human PIWI (gi:24431985) (PIWI start - 731), human AGO1 (gi:6912352)(PIWI start - 575) and human AGO2 (gi: 29171734)(PIWI start - 577) [25], with the AtAGOs and MtAGOs.

### Protein sequence alignment and phylogenetic tree building

The complete protein sequence of each putative AGO or DCL gene was used for the construction of the phylogenetic tree. Protein alignment was done using T-Coffee software [46-48]. The phylogenetic tree was generated with MEGA4.0 software [49] using the distance model for amino acid substitution of Jones-Taylor-Thornton (JTT) matrix, the Neighbor-joining algorithm for clustering and 1000 replications for the bootstrap analysis.

### RNA Extraction and quantitative Real Time PCR (qPCR)

Extraction of total RNA from the shoots and roots of four plants per treatment was done as previously described [4]. The RNA samples were treated with the TURBO DNA-free Kit (Ambion, Austin, Texas, USA) to eliminate DNA contaminations. Total RNA pools from shoots and roots and per treatment were made. The RNA quantification was performed using the NanoDrop 1000 Spectrophotometer (Thermo Scientific, Waltham, Massachusetts, USA). After DNAse digestion, the absorbance ratios of the RNA samples at 260/280 nm and 260/230 nm were between1.9-2.0. One μg of RNA from each pool was reverse transcribed using the Promega-ImProm-II™ Reverse Transcription System (Promega, Madison, Wisconsin, USA) according to the manufacturer's instructions, using the poly-T oligonucleotide primer. Three independent reverse-transcription reactions (RT) were performed using the RNA pools and each one was diluted 5-fold before each quantitative Real Time Polymerase Chain Reaction (qPCR) reaction.

PCR primers (Additional file 3) were designed using the Beacon Designer software (version 7.0) (Premier Biosoft International, Palo Alto, California, USA). Primers were designed to have a size between 18-24 bp, GC content of 40-60% and melting temperature (Tm) of 58-62°C. The MtAGO1 and MtDCL1 primer pairs were designed to amplify a region containing the cleavage site of miR168 and miR162 respectively. Other criteria, such as primer self-annealing, were also taken into account. Predicted fragment size ranged between 80 and 180 bp. Oligonucleotides were synthesized by Stabvida (Stabvida, Caparica, Portugal).

qPCR reactions were performed in an iQ™5 Real-Time PCR Detection System (Bio-Rad Laboratories, München, Germany), by adding 10μl of iQ™ SYBR Green Supermix (Bio-Rad Laboratories), 4μL of diluted cDNA, 0.5 pmol of each primer, and water to a final volume of 20μL. After one initial incubation step at 95°C for 3 min, amplifications were performed for 40 cycles with the following cycle profile: a denaturing step at 95°C for 15 s followed by an annealing step at 60°C for 10 s, and an extension step of 72°C for 10 s. Fluorescence data were collected during the 72°C step, and the specificity of qPCR products was confirmed by performing a melting temperature analysis at temperatures ranging from 55°C to 95°C in intervals of 0.5°C. PCR products were run in a 2.5% agarose gel to confirm the existence of a unique band with the expected size.

Reference genes were selected based on a previous study where the accumulation of HDA3, L2, APRT, ELF-1α, ACT7 and ACT11 (Additional file 3) was quantified on cDNAs from the plants with different water status and plant organs (shoots and roots) using the geNorm [50] and NormFinder [51] in Genex software (version 4.3.8) (MultiD, Göteborg, Sweden). L2 was found to be the best reference gene for the experimental conditions (Ct, MWD, SWD and Rec) and plant organs (shoots and roots) used in this work.

For all the genes studied, three independent cDNA samples of the RNA pools from each experimental condition were amplified in technical duplicates, giving a total of 6 replicates for each treatment. The raw, background-subtracted, fluorescence data provided by the iQ5 software (version 2.0) was analyzed by the real-time PCR Miner software (version 2.2) [52,53]. The resulting PCR efficiency and cycle number quantification were used for transcript quantification. The efficiency for each gene was calculated using the arithmetic mean of all efficiencies given by PCR Miner.

The Pfaffl method [54] was used for the relative quantification of the transcript accumulation of the genes of interest using L2 as reference gene. For each gene the results were normalized against the shoot control treatment. The One Way ANOVA Test of significance was used to compare the four conditions in each organ followed by the Tukey Test (SigmaStat version 3.5, Systat Software Inc., San Jose, California).

The Minimum Information for Publication of Quantitative Real Time PCR Experiments (MIQE) check list could be find in the Additional file 4[55].

### miR162 and miR168 northern blot analysis

Total RNA (15μg per lane) was blotted to a Hybond-NX membrane (GE Healthcare, Piscataway, NJ, USA) and hybridized according to Trindade et al [4]. Small nuclear RNA U6 was used as a loading control. The Locked Nucleic Acid (LNA)-modified oligonucleotides (Exiqon, Vedbaek, Denmark) complementary to miR168 and miR162 and the molecular weight probes were labeled with γP^32^-ATP (PerkinElmer, Waltham, Massachusetts, USA) according to Trindade et al [4]. Membranes were striped with boiling 0.1% SDS and hybridized with the small nuclear RNA U6 loading control probe.

## Results

### Molecular characterization of MtDCLs and MtAGOs

A BLASTn and tBLASTn search in *M. truncatula *genome databases, using *A. thaliana *DCL and AGO cDNA and protein sequences, identified three putative coding sequences for Dicer-like (MtDCLs) genes and twelve putative coding sequences for Argonaute (MtAGOs) genes (Table 1). MtAGO1 was identified in *M. truncatula *gene index database (MTGI9.0), whereas MtDCL2, MtDCL3 and MtAGO11 were only identified in *M. truncatula *annotated genome (Mt3.0) (Table 1).

**Table 1:** Characteristics of Dicer-like and Argonaute coding sequences and proteins identified in *M. truncatula*.

Gene Name	BAC	IMGAG Gene Loci	MTGI9.0 acession	Protein			**Chr**.	Genomic Region (start-end)	BLASTn BLASTp
							
				Size (a.a.)	MW (KDa)	pI (pH)			
**Dicer-like genes**									

MtDCL1	AC150443	Medtr7g146220.1	NP7270921 TC129362	1939	218.32	6.22	7	34948437-34935433	AtDCL1
MtDCL2	AC192958	Medtr2g129960.1		1416	160.96	7.30	2	31566180-31555830	AtDCL2
MtDCL3	AC137830	Medtr3g139020.1	NP7267858 NP7267870	1727	192.35	6.68	3	35653717-35640861	AtDCL3

**Argonautes**									

MtAGO1			TC126820	1046	116.02	9.47			AtAGO1
MtAGO12a	AC160838	Medtr8g118920.1		876	98.62	8.79	8	26846440-26851968	AtAGO10
MtAGO12b	AC231336	Medtr2g074590.1 Medtr2g074600.1 Medtr2g074610.1		732	83.56	8.37	2	17220707-17227158	AtAGO10
MtAGO12c	AC136450 AC231336	Medtr2g074570.1	NP7267711	520	59.61	9.99	2	17189793-17194311	AtAGO10
MtAGO2a	AC225510	Medtr4g114860.1	TC135942 TC116031 TC136095	916	103.54	9.03	4	26413990-26408838	AtAGO2
MtAGO2b	AC209534	Medtr2g034460.1		883	100.58	9.01	2	9642645-9638911	AtAGO2
MtAGO7	CU179907	Medtr5g045600.1	AW693202 BI309506	1016	116.65	9.44	5	19094952-19099268	AtAGO7
MtAGO4a	AC147429	Medtr3g111450.1	TC114668 TC126933	824	92.40	8.80	3	28346658-28352637	AtAGO4
									
MtAGO4b	AC131455	Medtr5g094930.1	TC114471	942	105.49	9.20	5	37632339-37642376	AtAGO4
MtAGO4c	AC131455	Medtr5g094940.1	TC112620	912	102.97	9.32	5	37643490-37650687	AtAGO4
MtAGO6	CU468297	Medtr3g105930.1		935	104.52	8.57	3	26600859-26609775	AtAGO6
MtAGO11	CT030192	Medtr3g016400.1		886	101.10	9.18	3	3169515-3175689	AtAGO4
		Medtr3g016410.1							
		Medtr3g016420.1							

BLASTn and BLASTp were performed with the MtAGOs and MtDCLs against the *A. thaliana *databases in NCBI [36]. BAC, Bacterial artificial chromosome accession number in Mt3.0; IMGAG, the International Medicago Genome Annotation Group; MTGI9.0, *Medicago truncatula *Gene Index (MTGI) 9.0 reference; MW, Molecular Weight; pI, Isoelectric point; Chr., Chromosome.

**Figure 1:**
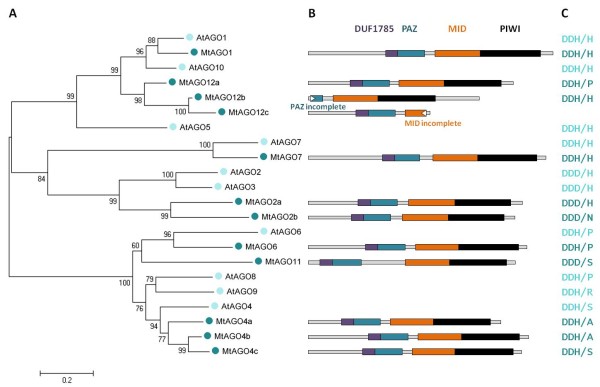
**Phylogenetic relations and characteristics of *Medicago truncatula *AGOs**. (A) Evolutionary relationship of *M. truncatula *and *A. thaliana *(At) AGOs. The complete protein sequences were aligned using the T-Coffee software [46-48] and a Neighbour joining tree was constructed using the MEGA4.0 software [49]. (B) Characterization of the MtAGO proteins domains. The protein domains were obtained using the Conserved Domains Database (CDD) database of NCBI. The Argonaute protein domains DUF1785 (purple), PAZ (dark-blue), MID (orange) and PIWI (black) are shown. (C) The catalytic center of MtAGOs was obtained from the alignment of the PIWI domains, corresponding to the positions of the aspartate, aspartate and histidine (DDH) motif and the Argonaute 1 histidine at position 800 (H800). D, aspartate, H, histidine, S, serine, A, alanine, P, proline, R, arginine. Figure B to scale.

The International Medicago Genome Annotation Group (IMGAG, Mt3.0) annotated MtAGO12b as three independent genes: Medtr2g074590.1, Medtr2g074600.1 and Medtr2g074610.1 (Additional file 5). But each sequence corresponded to an incomplete Argonaute gene. The Fgenesh annotation of the *M. truncatula *genome generates a unique gene sequence instead of the three incomplete genes. Therefore we decided to use the Fgenesh annotation since it retrieved a more complete Argonaute gene sequence. The region of Medtr2g074590 not considered by the annotation made by Fgenesh, presented several N entries. This could be the reason why the Paz domain is incomplete and the DUF1785 is missing (Figure 1, B, AGO12b). The same problem occurred with MtAGO11 that corresponds to the junction of: Medtr3g016400.1, Medtr3g016410.1 and Medtr3g016420 (Additional file 6).

**Figure 2:**
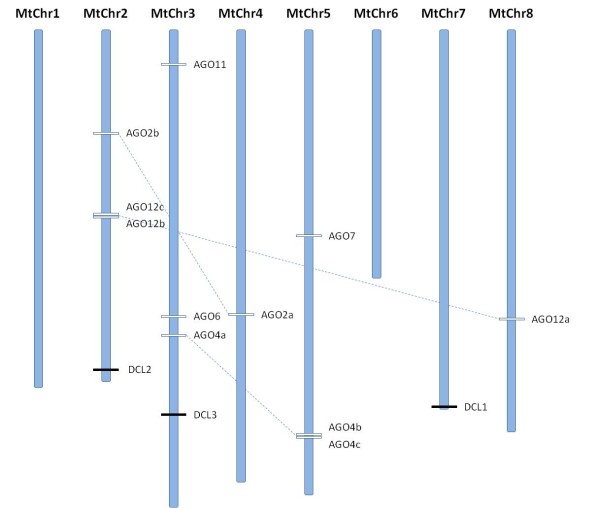
**DCLs and AGOs Loci in *M. truncatula *chromosomes (MtChr)**. The AGO1 locus is not annotated in the *M. truncatula *genome because is not totally sequenced.

The putative MtDCL genes probably encode proteins with molecular weights that range between 160.96 and 218.32 KDa, with a neutral isoelectric point ranging from 6.22 to 7.30 (Table 1). The predicted MtAGO proteins have a lower molecular size of ~100 KDa and a basic isoelectric point between 8.37 and 9.99 (Table 1). The identified DCL and AGO genes are distributed on chromosomes 2, 3, 4, 5, 7 and 8 of *M. truncatula *(Figure 2) but more concentrated in chromosomes 2, 3 and 5.

### *M. truncatula *DCL and AGO protein domains

**Figure 3:**
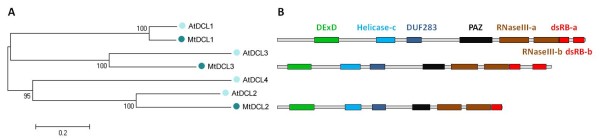
**Phylogenetic relations and characteristics of *Medicago truncatula *DCLs**. (A) Evolutionary relationship of *M. truncatula *and *A. thaliana *(At) DCLs. The complete protein sequences were aligned using the T-Coffee software [46-48] and a Neighbour joining tree was constructed using the MEGA4.0 software [49]. (B) Characterization of MtDCL proteins domains. The protein domains were obtained using the Conserved Domains Database (CDD) database of NCBI. The Dicer-like protein domains DExD (green), Helicase-c in (blue), DUF283 (dark-blue), PAZ (black), RNAase III (brown), dsRB (red) are shown. Figure B to scale.

To assign the putative *M. truncatula *DCL and AGO genes a Neighbor-joining phylogenetic tree was generated with the predicted complete protein sequences of *M. truncatula *and *A. thaliana *DCLs and AGOs (Figure 3, A and Figure 1, A). DCLs and AGOs clustered into 4 and 3 subgroups respectively, similar to those described by Margis et al, and Vaucheret [12,56]. The names of the *M. truncatula *predicted proteins were given according to their phylogenetic relationship with *A. thaliana *protein sequences.

The protein domains searches using the CDD software from NCBI revealed the presence of DExD, Helicase-c, DUF283, PAZ, RNaseIIIa/b and dsRBa/b in the predicted DCL protein sequences analyzed (Figure 3, B). MtDCL2 has only one dsRB domain, similar to the *A. thaliana*, *Oryza sativa *and *P. trichocarpa *DCL2 proteins [12].

Crystal structure of a full-length Argonaute protein, from the *archaea *species *Pyrococcus furious*, showed that the sequence motif originally defined as PIWI domain by Cerutti et al [57] consists of two structural domains, termed MID and PIWI [58]. Wang et al, [25] identified the amino acid that separates the MID domain from the PWI domain in *Thermus thermophilus *(Tt), *Pyrococcus furiosus *(*Pf*), *Aquifex aeolicus *(Aa) and human (Hs) AGO protein sequences [25]. The CDD software can only find the PIWI domain defined by Cerutti et al [57] and does not separates the MID and PIWI domains. We aligned these protein sequences together with MtAGOs and AtAGOs, to find the domains separation amino acid (Additional file 7).

Almost all predicted MtAGO proteins presented the domains DUF1785, PAZ, MID and PIWI (Figure 1, B). An exception to this is MtAGO12b where the DUF1785 and the PAZ domain are missing. There are several N entries upstream of the start codon, indicating that the sequence quality at that site is not good. MtAGO12c contains one incomplete MID domain and lacks the PIWI domain, but this fact is unexplainable.

Several structural studies have shown that the PIWI domain folds similar to RNaseH proteins [58]. Consistent with this observation, some plant and animal Argonaute proteins are known to cleave the target mRNAs that have sequence complementary to the small RNAs [19,59]. The catalytic center of these proteins are known to possess three conserved metal chelating amino acid residues in the PIWI domain i.e. aspartate, aspartate and histidine (DDH) that function as a catalytic triad. In *A. thaliana *AGO1 the histidine at position 800 (H800) was also shown to be critical for this endonuclease activity [19].

To interrogate which of the predicted MtAGOs included the conserved catalytic residues and could potentially act as the slicer component of the silencing effectors complexes, we aligned the PIWI domains of all the predicted MtAGOs and AtAGOs using T-Coffee (Additional file 7). Two predicted protein sequences, MtAGO1 and MtAGO7 were found to have the conserved domain DDH/H (Figure 1, C). In other MtAGOs like MtAGO12b the motif was missing or the residue H800 substituted by A, S or P, or in MtAGO2 the H (in the DDH motif) is substituted by one D (shifting to a DDD motif), characteristic of AGO2 and AGO3 proteins in *A. thaliana *and *O. sativa *[18].

### qPCR of the MtDCLs and MtAGOs

**Figure 4:**
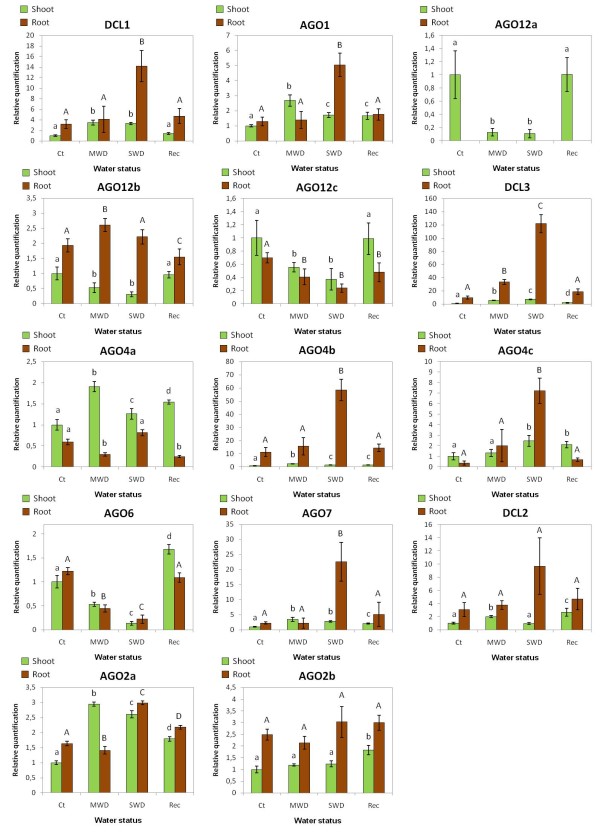
**Relative accumulation of Dicer-like and Argonautes mRNAs in *M. truncatula *in different water status**. The shoots (green) and roots (brown) of *M. truncatula *were the organs analyzed in the different water treatment conditions imposed. Values are the mean of two technical replicates of three independent cDNAs for each treatment and bars represent standard errors. The relative mRNA accumulation was calculated using L2 as the reference gene and normalized against the shoot control treatment. The AGO1 and DCL1 primer pair was designed to give one amplicon with the cleavage site of their corresponding miRNA, miR168 and miR162 respectively. A One Way ANOVA Test of significance was used to compare the four conditions in each organ followed by the Tukey Test (p-value <0.05). Ct, Control; MWD, Moderate Water Deficit; SWD, Severe Water Deficit, Rec, Recovery.

Plants have several Dicer-like and Argonaute genes with different functions. AtDCL1, AtAGO1 and AtAGO10 are involved in the miRNAs production and function [20,56]. In our study, plants under water deficit increased the transcript levels of MtDCL1 and MtAGO1 in the roots and shoots (Figure 4). Notably, in roots, 5 and 3.5 fold increase was found for MtDCL1 and MtAGO1 transcripts accumulation under severe water deficit.

Shoots of plants subjected to water deprivation showed a decrease in the transcript abundance of MtAGO12a, MtAGO12b and MtAGO12c (Figure 4). However a different picture was seen in roots: the mRNA of MtAGO12a was not detected; MtAGO12b maintained its mRNA level under water deficit; and the level of MtAGO12c transcripts decreased significantly following the same pattern found in shoots.

AtDCL3 cleaves endogenous dsRNA producing 24-nt sRNAs and AtAGO4, AtAGO6 and AtAGO9 use these sRNAs to direct transcriptional gene silencing (TGS), which perform chromatin remodeling [56]. MtAGO6 was down regulated under water deficit in shoots and roots (Figure 4 andAdditional file 8). MtDCL3, MtAGO4b and MtAGO4c increased their transcript abundance in similar way under water deficit in both shoots and roots. The mRNA levels of MtAGO11 (a protein similar to AtAGO6 - Figure 1, A) could not be quantified.

AtAGO7 is involved in the biogenesis of trans-acting small RNAs (ta-siRNAs) derived from TAS3 RNA [56]. Both shoots and roots presented an increase in transcript levels of MtAGO7 under water deficit with a very high variation in severe water deficit in the roots (Figure 4 andAdditional file 8).

In Arabidopsis DCL2 cleaves double-stranded virus RNA producing 22nt small RNAs [56]. A small but significant variation was found for the accumulation of MtDCL2 transcripts in shoots under MWD condition. In the roots no significant variation was found along the water deficit treatments and recovery (Figure 4).

The function of AtAGO2 is not clear but has a distinct characteristic from other AGOs, it is highly specific for small RNAs with a 5' terminal adenosine [26]. Under water deficit MtAGO2a transcripts levels increased while MtAGO2b remained almost stable in both shoots and roots (Figure 4).

### Expression of miR162 and miR168a/b and their targets during water deficit

**Figure 5:**
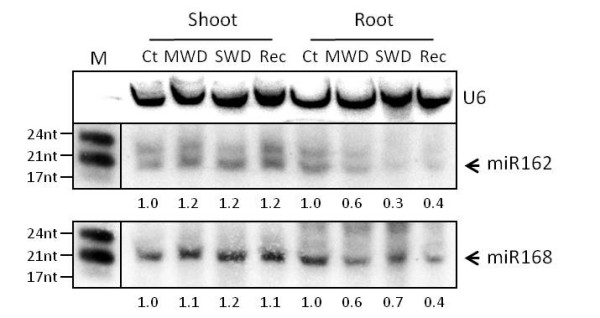
**miR162 and miR168 expression in shoots and roots of *M. truncatula *in different water status**. The U6 small nuclear RNA was used as internal loading control. The accumulation of miR162 and miR168 (numbers indicated under each lane) was quantified according to U6 small nuclear RNA loading control and normalized to control conditions. The membrane was first hybridized with miR162 probe and then striped and rehybridized with miR168 probe. (M) miRNA size marker with three bands of 24, 21 and 17 nt (New England Biolabs) is shown in the left.. Ct, Control; MWD, Moderate Water Deficit; SWD, Severe Water Deficit; Rec, Recovery.

DCL1 and AGO1 are two enzymes that have very important roles in miRNA maturation and functionality. In *M. truncatula *their mRNAs are targeted by miR162 and miR168 respectively [2,3]. In *M. truncatula *miR162 and miR168 are expressed in different plant organs (Additional file 9). For miR162, two bands were visible (Figure 5 andAdditional file 9): one band of 21-nt that correspond to the miR162 size [2,3] while the other low intensity band is of 24-nt. For miR168, again two bands are visible, one of 21-nt that corresponds to the miRNA [2,3] and a faint band of 24-nt. The probable reason for the extra bands is that DCL3 competes with DCL1 for the same miRNA precursors to produce small RNAs molecules with 24-nt [60].

The expression of both miR168 and miR162 did not seem to change in the shoots of plants subjected to water deficit and in the recovered plants when compared with the controls (Figure 5). On the other hand both miRNAs decrease their accumulation in roots subjected to water deficit and their expression did not returned to the control levels when plants were re-watered. It seems that only in the roots and especially for the MtDCL1 a post transcriptional control mediated by miRNAs is taking place (Figure 4 and 5).

## Discussion

In the present work we have identified 3 putative DCL and 12 putative AGO genes in the genome of *M. truncatula *from which only the transcript levels of MtAGO11 could not be detected. Thus far the identification and characterization of these important gene families was mostly limited to *A. thaliana *and *O. sativa*.

### The catalytic center of MtAGOs

The slicing activity has been demonstrated for *A. thaliana *AGO1, AGO2, AGO4 and AGO7 [19,22,61,62] and almost all of them have a catalytic center carrying a DDH motif also found in animal AGOs [24]. The exception is AtAGO2, which has a DDD motif (Figure 1, C) [19]. In *Homo sapiens *the AGO3 protein has a DDH motif but without a slicing activity [59,63]. On the other hand, the *Drosophila melanogaster *PIWI domain has one DDK motif and has catalytic activity [64]. In conclusion, the existence or absence of a DDH motif does not necessarily imply a slicing activity. Baumberger and Baulcombe [19] showed that the histidine residue in position 800 is essential for the slicing activity of AtAGO1. However AtAGO4 has a serine residue instead of a histidine in position 800 but still has slicing activity. Therefore the existence of this residue in Argonautes may not be an obligatory determinant for their cleavage activity. The DDH/H or DDD/H motifs are present in MtAGO1, MtAGO2a and MtAGO7 (Figure 1, C) and are homologous to AtAGO1, AtAGO2 and AtAGO7, indicating that they probably have slicing activity in *M. truncatula*. It is also possible that MtAGO4s, MtAGO6, MtAGO11, MtAGO12a and MtAGO12 presenting a DDH/(A/S/P) motif and MtAGO11 and MtAGO2b presenting a DDD/S motif may have as well a slicing activity.

### MtDCL1

MtDCL1 mRNA levels increased in *M. truncatula *under water deficit (Figure 4), which may imply the increase of mature miRNAs. DCL1 is subjected to negative feedback regulation by miR162 [27]. In our case, miR162 is less accumulated in the roots under water deficit, having the lowest accumulation in SWD (Figure 5). The correlation of this with the increase of MtDCL1 transcript levels, most notorious in roots under water deficit (Figure 4), probably indicates that the regulation of MtDCL1 by miR162 is relaxed in response to water deprivation, increasing the possibility of a higher DCL1 activity in plants subjected to this stress condition. Since DCL1 is involved in synthesis of miRNAs, this suggests that these sRNAs may play an important role in plant responses or adaptation to water deficit.

### MtAGO1

AGO1 is the main protein mediating miRNA post-transcriptional directed regulation and *ago1 *mutants show several developmental defects [28]. The AGO1 homeostasis is maintained by the post-transcriptional regulation of AGO1 by miR168 and the stabilization of miR168 levels by AGO1 [65]. Another way of regulating AGO1 is through AGO1-derived short interfering RNAs (siRNAs). However, for this type of regulation to happen it is required that these siRNAs were produced by DCL2 and DCL4 [66]. Three enzymes, RNA-dependent RNA polymerase (RDR6), Suppressor of gene silencing 3 (SGS3) and Silencing Defective 5 (SDE5) are involved in double strand RNA (dsRNA) production from the cleaved mRNA of AGO1. In addition Mallory et al. [67] demonstrated that AGO10 is a negative regulator of AGO1 levels and Brodersen et al [20] showed that AGO10 together with AGO1 mediate the translational repression of miRNAs targets in a miRNA-dependent manner.

In our case we observe that the transcript levels of MtAGO1 increased in *M. truncatula *under water deficit (Figure 4). However we could not correlate this with the variation of miR168 accumulation (Figure 5). Vaucheret et al. [28] verified that the over-accumulation of AGO1 causes developmental defects in Arabidopsis which means that the homeostasis of AGO1 is important to stabilize the functioning of the miRNA pathway. More evidence is needed to understand the MtAGO1 transcript increase in water deprived *M. truncatula *plants although this indicates an implement of the activity of miRNAs.

### MtAGO12a, MtAGO12b and MtAGO12c

MtAGO12a, MtAGO12b and MtAGO12c are similar to AtAGO10 as shown by BLASTn and BLASTp (Table 1) and have the highest homology with AtAGO10 (Figure 1, A). In *A. thaliana*, AGO10 promote the translation repression of some miRNAs targets [20]. Giving these homologies MtAGO12 enzymes probably share the same functionalities with AtAGO10. In *M. truncatula *shoots MtAGO12b and MtAGO12c transcript levels decreased in response to water deficit, suggesting that the mechanism of translation repression mediated by MtAGO12s is probably being shut down. In the roots these genes are differentially expressed, suggesting that they could have the same function but have evolved to respond differentially to water deficit, in a similar way to what was observed with the rice OsAGO1a-d under different stress conditions [18].

### MtAGO7

Argonaute 7 specifically associates with miR390 and directs the cleavage at the 3' end of its non-coding target TAS3 RNA [22,68]. The TAS3 cleavage products are stabilized by Suppressor of Gene Silencing 3 (SGS3), and one of the two TAS3 cleavage products is converted to dsRNA by RNA dependent RNA Polymerase 6 (RDR6). Finally this dsRNA is diced by DCL4 into 21-nt trans-acting siRNAs (ta-siRNAs) a process assisted by a dsRNA binding protein 4 (DRB4). The bioinformatic search for DCLs in *M. truncatula*, could not find a homolog sequence to *A. thaliana *DCL4, possibly because the *M. truncatula *genome is not yet fully sequenced. Nevertheless, three annotated genes homologous to the Auxin Response Factor 3 (ARF3) of *A. thaliana *were identified in the *M. truncatula *genome by Jagadeeswaran et al. [3] as targets of two TAS3-derived ta-siRNAs.

*A. thaliana *AGO7 mutants accelerate the juvenile to adult transition but not the onset of reproductive competence or flowering time [69]. ARF3 over-expression resulted in further acceleration of phase change and severe morphological and patterning defects of leaves and floral organs [70]. In our study we observed that MtAGO7 mRNA levels increased under water deficit in both shoots and roots (Figure 4). Several previous works correlated the role of TAS3 derived ta-siRNAs with plant development processes, but our results indirectly suggest that ARF3 can have a role in plant reaction to water deficit, maybe by repressing the development processes in the plants that are under stress conditions. This correlates with the observed reduction in growth and development of *M. truncatula *plants subjected to water deprivation when compared with the controls. The expression levels of the TAS3, the three ARF3 genes and TAS3 derived ta-siRNAs from *M truncatula *should be analyzed under water deficit to test this hypothesis.

### Water deficit response in *M. truncatula *and chromatin rearrangements

In plants, chromatin gene silencing involves the coordinated action of: (1) a DNA dependent RNA polymerase IV (Pol IVa and Pol IVb) that transcribes genomic regions with transposons and highly repeated sequences [71,72]; (2) a RNA dependent RNA polymerase 2 (RDR2), that produces a dsRNA sequence from the regions transcribed by RNA Pol IV [73]; (3) a Dicer-like 3 (DCL3), that cleaves the dsRNA in 24-nt sRNAs [10,61,73]; (4) the Argonaute 4 and 6, that are important for the accumulation of specific heterochromatin-related siRNAs, and for DNA methylation and subsequent transcriptional gene silencing [73-75].

We showed that the *M. truncatula *MtDCL3, MtAGO4b and MtAGO4c transcript levels increased under water deficit situations (Figure 4). However MtAGO4a had no clear response to water deprivation while MtAGO6 mRNA levels decreased. These results might suggest that the chromatin rearrangements may have a role in the plant responses to water deficit. A possible explanation for the differences in the transcript abundance patterns of the MtAGO4s (Figure 4) is that MtAGO4b and MtAGO4c are phylogenetically more related to each other than with MtAGO4a (Figure 1).

Havecker et al. [21] verified that in Arabidopsis AGO6 is only expressed in the shoot and root growing tips and AGO4 is expressed predominantly in leaves of adult plants and in all flower and embryo developmental stages. Both Argonautes bind to 5' adenosine 24-nt small RNAs meaning that these characteristics do not distinguish their functional diversification. The functional divergence between AGO4 and AGO6 is related with their expression in different tissues and the epigenetic modifications are influenced by interactions between the AGO protein and the different target loci [21]. We identified 3 genes as AGO4 and one as AGO6 in *M. truncatula *(Figure 1). These genes are possibly expressed in different tissues and have different degrees of interaction for the same loci as well. The increased MtDCL3, MtAGO4b and MtAGO4c mRNA levels under water deficit are very similar in both shoots and roots (Figure 4). Thus it is possible that the spatial production of small RNAs by MtDCL3 coincides with the spatial expression of MtAGO4b and MtAGO4c since they are related with the same mechanism of silencing.

## Conclusion

We identified and characterized three DCLs and twelve AGOs genes in the genome of *M. truncatula*. These genes probably encode enzymes that integrate different sRNA pathways and their transcript levels are modulated in response to water deficit. This modulation is more evident in roots. The processing and activation of miRNAs are up regulated as well as the sRNAs mediated DNA methylation mechanisms and the production of trans-acting small interfering RNAs. Our observations opened an opportunity to study the impact of sRNA metabolism in the response of legumes toward water deficit.

## Authors' contributions

CC and DMS conceived the design of the study. CC carried out all the experimental work. CC performed all the bioinformatics work: search and characterization of the AGO and DCL genes in *M. truncatula *genome, the search of the protein sequence domains using CDD, the protein sequence alignment and the phylogenetic tree building. CC and JAPP designed the qPCR experiments. CC carried out the qPCR experimental work. CC analyzed the qPCR data and performed statistical analysis of the results. CC wrote the paper. PF was involved in the writing and corrections of the manuscript. All authors read and approved the final manuscript.

## Supplementary Material

Additional file 1 **Scheme showing the water regime imposed to *M. truncatula *plants**. The average of the relative water content (RWC) of each experimental group is shown. Click here for file

Additional file 2 ***A. thaliana *Dicer-like and Argonaute sequences used for identification of DCLs and AGOs in *M. truncatula***. The Arabidopsis Information Resource (TAIR) accession number of the genes and their mRNA and protein accession numbers in NCBI database are shown. Click here for file

Additional file 3 **The Minimum Information for Publication of Quantitative Real Time PCR Experiments (MIQE) check list**. A complete list of all the procedures used in the qPCR experiment. Click here for file

Additional file 4 **Primers used for the quantification of transcript accumulation by qPCR in *M. truncatula***. Indication of the amplification product size (Amplicon size) and the PCR efficiency used for each pair of primers obtained from real-time PCR Miner software (version 2.2). The efficiency for each gene was calculated doing the arithmetic mean of all efficiencies given by PCR Miner. Click here for file

Additional file 5 **Annotation of MtAGO12b gene in *M. truncatula *genome using IMGAG (Mt3.0) and Fgenesh software**. IMGAG gives three independent annotated sequences (Medtr2g074590.1, Medtr2g074600.1 and Medtr2g074610.1) on the other hand Fgenesh annotates them as only one sequence. The image was obtained in the Medicago GBrowse from J. Craig Venture Institute [39]. Click here for file

Additional file 6 **Annotation of MtAGO11 gene in *M. truncatula *genome using IMGAG (Mt3.0) and Fgenesh software**. IMGAG annotates three independent annotated sequences (Medtr3g016400.1, Medtr3g016410.1 and Medtr3g016420) while Fgenesh annotates them as only one sequence. The image was obtained in the Medicago GBrowse from J. Craig Venture Institute [39]. Click here for file

Additional file 7 **Amino acid alignment of the PIWI domains of *M. truncatula *(Mt) and *A. thaliana *(At) Argonaute proteins**. The protein sequences were aligned using T-Coffee software [46-48]. The amino acids residues corresponding to the conserved aspartate, aspartate and histidine (DDH) catalytic triad residues are marked in black, while the *A. thaliana *Argonaute 1 histidine in the position 800 (H800) is in yellow. Amino acid positions corresponding to the beginning and end of the PIWI Domains in each protein are mentioned. TtAGO, *Thermus thermophilus*-0026 Argonaute (gi:46255097); PfAGO, *Pyrococcus furiosus*-0537 Argonaute (gi:18976909); AaAGO *Aquifex aeolicus*-1447 Argonaute (gi:15606619); HsPIWI, human PIWI (gi:24431985); HsAGO1, human Argonaute1 (gi:6912352); HsAGO2, human Argonaute2 (gi:29171734). Click here for file

Additional file 8 **Relative accumulation of MtDCL3, MtAGO4b, MtAGO4c, MtAGO7 mRNAs in *M. truncatula***. The shoots of *M. truncatula *plants were analyzed in the different water treatment conditions imposed to the plants. Values are the mean of two technical replicates of three independent cDNAs for each treatment and bars represent standard errors. The relative mRNA accumulation was calculated using L2 as the reference gene and normalized against the shoot control treatment. A One Way ANOVA Test of significance was used to compare the four conditions in each organ followed by the Tukey Test (p-value <0.05).. Ct, Control; MWD, Moderate Water Deficit; SWD, Severe Water Deficit, Rec, Recovery. Click here for file

Additional file 9 **Expression of miR162 and miR168 in various organs and seedling phase of *M. truncatula *plants**. Northern-blot analysis of shoots (S), roots (Rt), 8-day-old seedlings (Sd), young immature seed pods (Sp) and flowers (F) of *M. truncatula *plants in control conditions. The small nuclear RNA U6 was used as internal loading control for quantification of RNA gel blot signals which were normalized against the shoot samples (numbers indicated under each lane). The membrane was first hybridized with miR168 probe and then striped and rehybridized with miR162 probe. The molecular marker (M) is shown in the left and present three different sizes: 17 nt, 21 nt and 24nt. Click here for file
