# Supplementary figures and images for: In Medicago truncatula, water deficit modulates the transcript accumulation of components of small RNA pathways
Source: BMC Plant Biol. 2011 May 10;11:79. doi: 10.1186/1471-2229-11-79 (PMC3098777; doi:10.1186/1471-2229-11-79)

*Medicago truncatula*

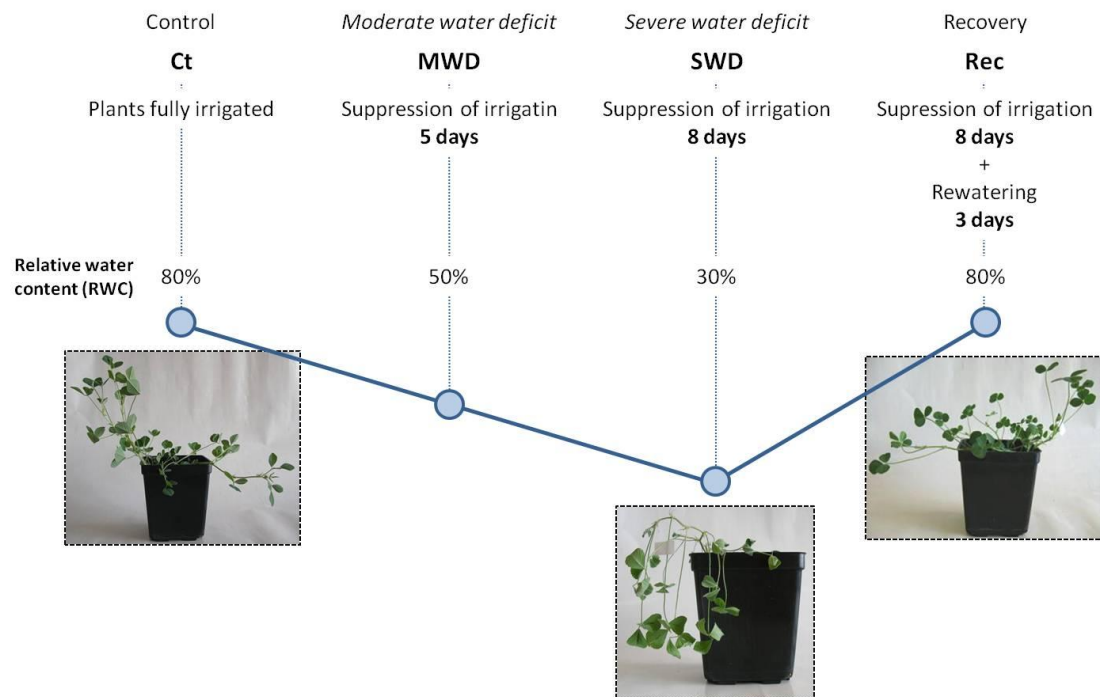

Supplement: Additional file 1 — Scheme showing the water regime imposed to M. truncatula plants. The average of the relative water content (RWC) of each experimental group is shown. [file 1471-2229-11-79-S1.PDF]

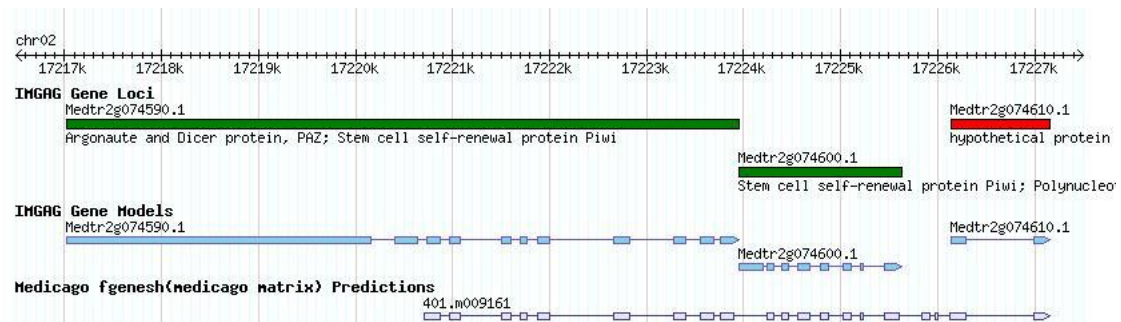

Supplement: Additional file 5 — Annotation of MtAGO12b gene in M. truncatula genome using IMGAG (Mt3.0) and Fgenesh software. IMGAG gives three independent annotated sequences (Medtr2g074590.1, Medtr2g074600.1 and Medtr2g074610.1) on the other hand Fgenesh annotates them as only one sequence. The image was obtained in the Medicago GBrowse from J. Craig Venture Institute [39]. [file 1471-2229-11-79-S5.PDF]

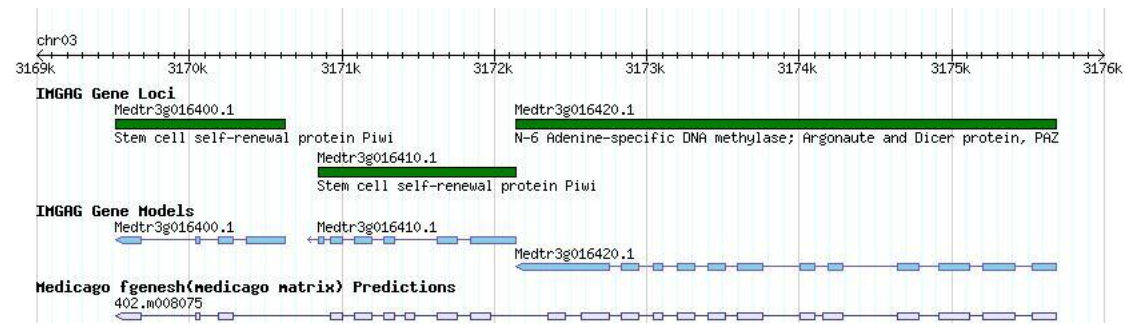

Supplement: Additional file 6 — Annotation of MtAGO11 gene in M. truncatula genome using IMGAG (Mt3.0) and Fgenesh software. IMGAG annotates three independent annotated sequences (Medtr3g016400.1, Medtr3g016410.1 and Medtr3g016420) while Fgenesh annotates them as only one sequence. The image was obtained in the Medicago GBrowse from J. Craig Venture Institute [39]. [file 1471-2229-11-79-S6.PDF]

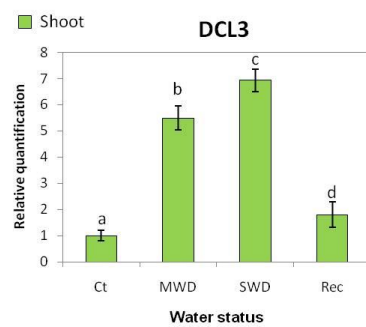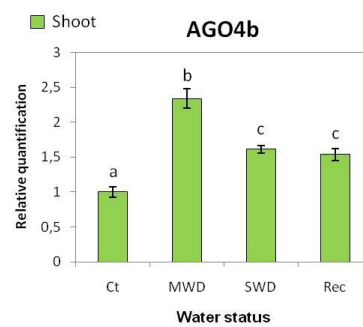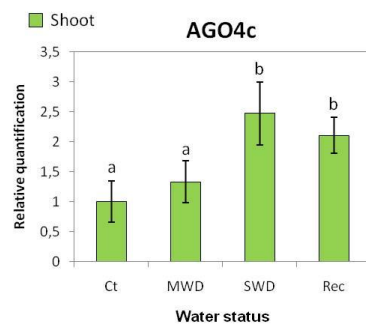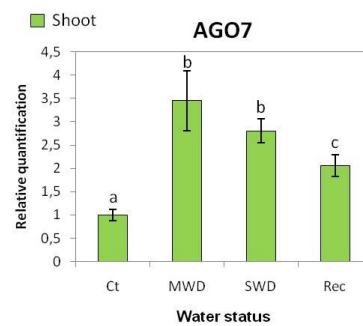

Supplement: Additional file 8 — Relative accumulation of MtDCL3, MtAGO4b, MtAGO4c, MtAGO7 mRNAs in M. truncatula. The shoots of M. truncatula plants were analyzed in the different water treatment conditions imposed to the plants. Values are the mean of two technical replicates of three independent cDNAs for each treatment and bars represent standard errors. The relative mRNA accumulation was calculated using L2 as the reference gene and normalized against the shoot control treatment. A One Way ANOVA Test of significance was used to compare the four conditions in each organ followed by the Tukey Test (p-value <0.05).. Ct, Control; MWD, Moderate Water Deficit; SWD, Severe Water Deficit, Rec, Recovery. [file 1471-2229-11-79-S8.PDF]

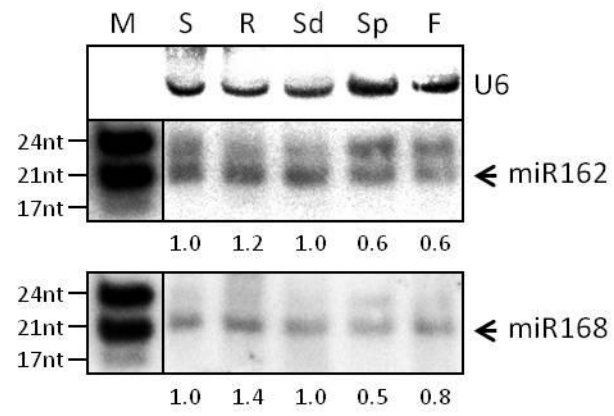

Supplement: Additional file 9 — Expression of miR162 and miR168 in various organs and seedling phase of M. truncatula plants. Northern-blot analysis of shoots (S), roots (Rt), 8-day-old seedlings (Sd), young immature seed pods (Sp) and flowers (F) of M. truncatula plants in control conditions. The small nuclear RNA U6 was used as internal loading control for quantification of RNA gel blot signals which were normalized against the shoot samples (numbers indicated under each lane). The membrane was first hybridized with miR168 probe and then striped and rehybridized with miR162 probe. The molecular marker (M) is shown in the left and present three different sizes: 17 nt, 21 nt and 24nt. [file 1471-2229-11-79-S9.PDF]
