# Supplementary material for: In Medicago truncatula, water deficit modulates the transcript accumulation of components of small RNA pathways
Source: BMC Plant Biol. 2011 May 10;11:79. doi: 10.1186/1471-2229-11-79 (PMC3098777; doi:10.1186/1471-2229-11-79)
Supplement: Additional file 2 — A. thaliana Dicer-like and Argonaute sequences used for identification of DCLs and AGOs in M. truncatula. The Arabidopsis Information Resource (TAIR) accession number of the genes and their mRNA and protein accession numbers in NCBI database are shown. [file 1471-2229-11-79-S2.PDF]

Gene name	Gene Abbreviation	TAIR	mRNA acession number	Protein accession number
**Dicer-like**				
**DCL1**	DCL1	AT1G01040	NM_099986.3	NP_171612.1
**DCL2**	DCL2	AT3G03300	NM_111200.5	NP_566199.4
**DCL3**	DCL3	AT3G43920	NM_001161191.1	NP_001154663.1
**DCL4**	DCL4	AT5G20320	NM_122039.4	NP_197532.3
**Argonaute**				
**AGO1**	AGO1	AT1G48410	NM_179453.2	NP_849784.1
**AGO2**	AGO2	AT1G31280	NM_102866.2	NP_174413.2
**AGO3**	AGO3	AT1G31290	NM_102867.1	NP_174414.1
**AGO4**	AGO4	AT2G27040	NM_128262.3	NP_565633.1
**AGO5**	AGO5	AT2G27880	NM_179779.2	NP_850110.1
**AGO6**	AGO6	AT2G32940	NM_128854.3	NP_180853.2
**AGO7**	AGO7	AT1G69440	NM_105611.3	NP_177103.1
**AGO8**	AGO8	AT5G21030	NM_122111.2	NP_197602.2
**AGO9**	AGO9	AT5G21150	NM_122122.2	NP_197613.2
**AGO10**	AGO10	AT5G43810	NM_123748.2	NP_199194.1
