# Supplementary material for: In Medicago truncatula, water deficit modulates the transcript accumulation of components of small RNA pathways
Source: BMC Plant Biol. 2011 May 10;11:79. doi: 10.1186/1471-2229-11-79 (PMC3098777; doi:10.1186/1471-2229-11-79)
Supplement: Additional file 3 — The Minimum Information for Publication of Quantitative Real Time PCR Experiments (MIQE) check list. A complete list of all the procedures used in the qPCR experiment. [file 1471-2229-11-79-S3.PDF]

Gene	Side	Primer sequence	Amplicon size	Efficiency
**Primers of the genes used in the screen of qRT-PCR reference(s) gene(s) selection**				
**Actin 11**	Forward	TTGGCATCACACCTTCTAC	158	0.81825
	Reverse	CCTGAATAGCAACATACATAGC		
**Actin 7**	Forward	GACAATGGAACTGGAATGG	196	0.8726
	Reverse	CAATACCGTGCTCAATGG		
**Aprt**	Forward	ATGTGCTGCGATTAAACTAC	139	0.9005
	Reverse	GGCTCCTTCTCCTTCAAC		
**Elf-1a**	Forward	ATTACCATTGATATTGCTTTGTG	186	0.87855
	Reverse	CTGTCCATCCTTAGAGATACC		
**L2**	Forward	GCTTACCACAAGTTCAGAG	164	0.90345
	Reverse	GCAATGAGACCAACCTTC		
**HDT3**	Forward	GCCAAGCACAGTGCCAAG	123	0.97335
	Reverse	CCATCAGCCAGTTTTCCTAAATC		
**Dicer-like genes**				
**MtDCL1**	Forward	CCATAAAGTGCGAGAATGTAGG	160	0.90525
	Reverse	AAGACAAGAGACGCCTTCG		
**MtDCL2**	Forward	AGACTCGGTGCTGGACTATC	170	0.91165
	Reverse	GCTCTTGAGAGGCATGAAGG		
**MtDCL3**	Forward	ATATGCGGTGAGCTGTCATC	116	0.9348
	Reverse	GATCTGTTCCCAGCTTGTGT		
**Argonaute genes**				
**MtAGO1**	Forward	GTGGAAGAGGTGGATATAGC	134	0.8695
	Reverse	GTAGGGAGCAGAAGATACAG		
**MtAGO12a**	Forward	GCTCAGCCTCACCGTGAAG	127	0,8194
	Reverse	TTTGTCCAGTTGCCCTCTTAAAC		
**MtAGO12b**	Forward	GGTTACCAACCTCCGGTTAC	104	0.9793
	Reverse	TTCCCACTCCTATCCATGCT		
**MtAGO12c**	Forward	GCGAATAGGCTTCCTGTGTATG	145	0,8928
	Reverse	TTCCCACCTTAAATTCTTGTTCCC		
**MtAGO2a**	Forward	GGCGAGGAGTTAAAAGATTTG	146	0,8830
	Reverse	GGGAACACATTTCCACTGG		
**MtAGO2b**	Forward	GGCAGAAGAATGGAGTCAGG	115	0,8619
	Reverse	TGGAATGCTCTCTTCAAATCAAG		
**MtAGO7**	Forward	CTCGCTTCCTTCTCAATTACC	137	0.95545
	Reverse	GCCAGTTCATGCTACCAAC		
**MtAGO4a**	Forward	AGTCCAGACAGAAGCCACAAGAG	95	0,8918
	Reverse	TCCACAACTTTGAAGCAGAGGTTC		
**MtAGO4b**	Forward	AAGCGACCTACATTTGTCCC	89	0.9733
	Reverse	GAGGATCTCTGGAGTGTGGA		
**MtAGO4c**	Forward	ACAAGCAGACCAACACATTATC	176	0.89955
	Reverse	ATGAACTGTCCAACTTGAGATG		
**MtAGO6-**	Forward	AAGGAAGTGTCTGAGTGATGTTG	151	0,8693
	Reverse	AAGATGCCCAGAATGCTCTATTG		
**MtAGO11**	Forward	GCCTTGAGAGGTCAGGAATC	175	
	Reverse	GGAAGCCACGACAACATTG		
