# Supplementary material for: In Medicago truncatula, water deficit modulates the transcript accumulation of components of small RNA pathways
Source: BMC Plant Biol. 2011 May 10;11:79. doi: 10.1186/1471-2229-11-79 (PMC3098777; doi:10.1186/1471-2229-11-79)
Supplement: Additional file 4 — Primers used for the quantification of transcript accumulation by qPCR in M. truncatula. Indication of the amplification product size (Amplicon size) and the PCR efficiency used for each pair of primers obtained from real-time PCR Miner software (version 2.2). The efficiency for each gene was calculated doing the arithmetic mean of all efficiencies given by PCR Miner. [file 1471-2229-11-79-S4.PDF]

EXPERIMENTAL DESIGN
Definition of experimental and control groups
Number within each group
Assay carried out by core lab or investigator's lab?
Acknowledgement of authors' contributions
**SAMPLE**
Description
Volume/mass of sample processed
Microdissection or macrodissection
Processing procedure
If frozen - how and how quickly?
If fixed - with what, how quickly?
Sample storage conditions and duration (especially for FFPE samples)

Medicago truncatula Gaertn. cv. Jemalong growth conditions are provided in the material and method section. The water status and physiological conditions of the different experimental groups were described in Nunes et al (2008) [34].

Eight-weeks-old plants were divided into 4 groups:

1. Control (Ct) group - plants fully irrigated (maximum soil water capacity) (relative water content (RWC) = 80%);
2. Moderate Water Deficit (MWD) group - plants subjected to water deprivation for 5 days (RWC = 50%);
3. Severe Water Deficit (SWD) group - plants subjected to water deprivation for 8 days (RWC = 30%);
4. Recovery (Rec) group – plants subjected to water deprivation for 8 days (RWC = 30%) and re-watered during 3 days (RWC = 80%).

The plants shoots and roots were separated and frozen separately in liquid nitrogen. The tissues were not processed immediately and were preserved at -80ºC. The samples were ground to dust using mortar and pestle. About 100 mg of tissue were used for extraction.

34. Nunes C, Araújo SDS, Silva JM, Fevereiro MPS, Silva AB: **Physiological responses of the legume model Medicago truncatula cv. Jemalong to water deficit**. *Environmental and Experimental Botany* 2008, **63**:289-296.

NUCLEIC ACID EXTRACTION
Procedure and/or instrumentation
Name of kit and details of any modifications
Source of additional reagents used
Details of DNase or RNAse treatment
Contamination assessment (DNA or RNA)
Nucleic acid quantification
Instrument and method
Purity (A260/A280)
Yield
RNA integrity method/instrument
RIN/RQI or Cq of 3' and 5' transcripts
Electrophoresis traces
Inhibition testing (Cq dilutions, spike or other)

Extraction of total RNA from the shoots and roots of four plants per treatment was done as previously described [4]. To remove possible DNA contamination, 10µg of RNA was treated with TURBO DNA-free Kit (Ambion, Austin, Texas, USA) according to the manufacturer's instructions. To confirm the complete DNA elimination from the samples a qPCR was performed using about 200ng of RNA. The primers used corresponded to the MtAGO1 gene (Additional file 4) and was used genomic DNA as positive control.

The RNA integrity was evaluated running the samples in a 2% agarose gel. The RNA quantification was performed using the NanoDrop 1000 Spectrophotometer (Thermo Scientific, Waltham, Massachusetts, USA). The absorbance ratios of the RNA samples at A260/A280nm were between 1.9 and 2.0. The RNA of the four plants per treatment was pooled.

4. Trindade I, Capitão C, Dalmay T, Fevereiro MP, Santos DMD: **miR398 and miR408 are up-regulated in response to water deficit in Medicago truncatula.** *Planta* 2010, **231**:705-16.

REVERSE TRANSCRIPTION
Complete reaction conditions
Amount of RNA and reaction volume
Priming oligonucleotide (if using GSP) and concentration
Reverse transcriptase and concentration
Temperature and time
Manufacturer of reagents and catalogue numbers
Cqs with and without RT
Storage conditions of cDNA

One µg of RNA from each pool was reverse transcribed using the Promega-ImProm-II™ Reverse Transcription System (Promega, Madison, Wisconsin, USA) according to the manufacturer's instructions, using the poly-T oligonucleotide primer. Three independent-reverse-transcription reactions (RT) were performed using the RNA pools. cDNA was stored in at -20°C.

**qPCR TARGET INFORMATION**
If multiplex, efficiency and LOD of each assay.
Sequence accession number
Location of amplicon
Amplicon length
In silico specificity screen (BLAST, etc)<br>Pseudogenes, retropseudogenes or other homologs?<br>Sequence alignment
Secondary structure analysis of amplicon
Location of each primer by exon or intron (if applicable)
What splice variants are targeted?

Multiplex qPCR was not performed. Sequence accession numbers are in the manuscript Table 1. The MtAGO1 and MtDCL1 primer pairs were designed to amplify a region containing the cleavage site of miR168 and miR162 respectively. The amplicon length is included in the Additional file 2. *In silico* screen were performed with NCBI Primer-Tool (http://www.ncbi.nlm.nih.gov/tools/primer-blast/) and CviT-Blastn in Mt3.0 (http://www.medicago.org/genome/blast.php). If possible the primers were designed close to the 3'end of the gene. No splice variants were targeted.

**qPCR OLIGONUCLEOTIDES**
Primer sequences
RTPrimerDB Identification Number
Probe sequences
Location and identity of any modifications
Manufacturer of oligonucleotides
Purification method

Primer sequences are included in the manuscript as Additional file 3. No modifications were used. Primers were purchase from Stabvida (Stabvida, Caparica, Portugal).

qPCR PROTOCOL
Complete reaction conditions
Reaction volume and amount of cDNA/DNA
Primer, (probe), Mg++ and dNTP concentrations
Polymerase identity and concentration
Buffer/kit identity and manufacturer
Exact chemical constitution of the buffer
Additives (SYBR Green I, DMSO, etc.)
Manufacturer of plates/tubes and catalog number
Complete thermocycling parameters
Reaction setup (manual/robotic)
Manufacturer of qPCR instrument

Each qPCR reaction had a 20 µl reaction volume containing:

cDNA corresponding to 10ng input RNA
250 nM of each forward and reverse primer
10 µl of iQ™ SYBR Green Supermix (Bio-Rad Laboratories, München, Germany)

Plates were purchased from VWR (Cat# 732-4924) and lids were purchased from BioRad (Cat# MSB1001).

Cycling parameters were:
1 Cycle
95°C for 3 min,
40 Cycles
95°C for 10 sec
65°C for 10 sec
72°C for 10 sec (Plate read)
1 Cycle
Melting curve from 55°C to 95°C, read every 0.5°C, hold 10 sec

Reactions were set up manually and qPCRs were performed with the iQ™5 Real-Time PCR Detection System (Bio-Rad Laboratories).

qPCR VALIDATION
Evidence of optimization (from gradients)
Specificity (gel, sequence, melt, or digest)
For SYBR Green I, Cq of the NTC
Standard curves with slope and y-intercept
PCR efficiency calculated from slope
Confidence interval for PCR efficiency or standard error
r2 of standard curve
Linear dynamic range
Cq variation at lower limit
Confidence intervals throughout range
Evidence for limit of detection
If multiplex, efficiency and LOD of each assay.

No optimization of the PCR protocol was performed. The specificity of the amplification products have been confirmed by size estimations on a 2.5% agarose gel and by analyzing their melting curves. The raw, background-subtracted, fluorescence data provided by the iQ5 software (version 2.0) was analyzed with the real-time PCR Miner software (version 2.2) [52, 53]. The resulting PCR efficiency and Cq values were used for transcript quantification. The efficiency for each pair of primers was calculated doing the arithmetic mean of all efficiencies given by PCR Miner.

52. Zhao S, Fernald RD: **Comprehensive algorithm for quantitative real-time polymerase chain reaction.** *Journal of computational biology : a journal of computational molecular cell biology* 2005, **12**:1047-64.
53. **Real-time PCR Miner** [www.ewindup.info/miner/version2].

DATA ANALYSIS
qPCR analysis program (source, version)
Cq method determination
Outlier identification and disposition
Results of NTCs
Justification of number and choice of reference genes
Description of normalisation method
Number and concordance of biological replicates
Number and stage (RT or qPCR) of technical replicates
Repeatability (intra-assay variation)
Reproducibility (inter-assay variation, %CV)
Power analysis
Statistical methods for result significance
Software (source, version)
Cq or raw data submission using RDML

Cq's were determined with the real-time PCR Miner software (version 2.2) [52, 53]. No data was excluded from the calculations. Reference genes were selected based on a previous study where the accumulation of HDA3, L2, APRT, ELF-1α, ACT7 and ACT11 (Additional file 3) was quantified on cDNAs from the different plant treatments and plant organs (shoots and roots) using the geNorm [50] and NormFinder [51] in Genex software (version 4.3.8) (MultiD, Göteborg, Sweden). L2 was found to be the best reference gene for the experimental conditions (Ct, MWD, SWD and Rec) and plant organs (shoots and roots) used in this work. The Pfaffl method was used for the relative quantification of the transcript accumulation of the genes of interest using L2 as reference gene [54]. For each gene the results were normalized against the shoot control treatment. The One Way ANOVA Test of significance was used to compare the four conditions in each organ followed by the Tukey Test (SigmaStat version 3.5, Systat Software Inc., San Jose, California). Number and stage (RT or qPCR) of technical replicates: 3 RTs for each pool and 2 qPCR repeats for each RT, giving a total of 6 qPCRs for each treatment/organ.

50. Vandesompele J, De Preter K, Pattyn F, Poppe B, Van Roy N, De Paepe A, Speleman F: **Accurate normalization of real-time quantitative RT-PCR data by geometric averaging of multiple internal control genes.** *Genome biology* 2002, **3**:RESEARCH0034.
51. Andersen CL, Jensen JL, Ørntoft TF: **Normalization of real-time quantitative reverse transcription-PCR data: a model-based variance estimation approach to identify genes suited for normalization, applied to bladder and colon cancer data sets.** *Cancer research* 2004, **64**:5245-5250.
52. Zhao S, Fernald RD: **Comprehensive algorithm for quantitative real-time polymerase chain reaction.** *Journal of computational biology* 2005, **12**:1047-1064.
53. **Real-time PCR Miner** [www.ewindup.info/miner/version2].
54. Pfaffl MW: **A new mathematical model for relative quantification in real-time RT-PCR.** *Nucleic acids research* 2001, **29**:e45.
