# Supplementary material for: In Medicago truncatula, water deficit modulates the transcript accumulation of components of small RNA pathways
Source: BMC Plant Biol. 2011 May 10;11:79. doi: 10.1186/1471-2229-11-79 (PMC3098777; doi:10.1186/1471-2229-11-79)
Supplement: Additional file 7 — Amino acid alignment of the PIWI domains of M. truncatula (Mt) and A. thaliana (At) Argonaute proteins. The protein sequences were aligned using T-Coffee software [46-48]. The amino acids residues corresponding to the conserved aspartate, aspartate and histidine (DDH) catalytic triad residues are marked in black, while the A. thaliana Argonaute 1 histidine in the position 800 (H800) is in yellow. Amino acid positions corresponding to the beginning and end of the PIWI Domains in each protein are mentioned. TtAGO, Thermus thermophilus-0026 Argonaute (gi:46255097); PfAGO, Pyrococcus furiosus-0537 Argonaute (gi:18976909); AaAGO Aquifex aeolicus-1447 Argonaute (gi:15606619); HsPIWI, human PIWI (gi:24431985); HsAGO1, human Argonaute1 (gi:6912352); HsAGO2, human Argonaute2 (gi:29171734). [file 1471-2229-11-79-S7.PDF]

```
AtAGO1    739 VLVDALSRRIPLV--SDRPTIIFGADVTHPHPGED-SSPSIAAVVA----SQDWPEITKYAG-LVCAQAHRQELIQDLFKEWKDPQ----KGVVTGGMIKELLIAFRRS-
MtAGO1    733 VLVDALSRRIPLV--SDRPTIIFGADVTHPHPGED-SSPSIAAVVA----SQDWPEITKYAG-LVCAQAHRQELIQDLFKQWQDPV----RGTLTGGMIKELLISFRRA-
AtAGO10   686 VLVDAISCRIPLV--SDIPTIIFGADVTHPENGEE-SSPSIAAVVA----SQDWPEVTKYAG-LVCAQAHRQELIQDLYKTWQDPV----RGTVSGGMIRDLLISFRKA-
MtAGO12a  580 VLLDAISCRIPLV--SDVPTIIFGADVSHPESGED-VCPSIAAVVA----SQDWPEVTKYAG-LVCAQPPREEIIKDLFKCWNDPR----RGIVYGGMIRELLLSFQKA-
MtAGO12b  298 VLLDALSWRIPLV--SDIPTIIFGADVTHAESGDD-SGPSIAAVVA----SQDWPEVTKYAG-LVCAQPHREELIQDLFKSWKDPQ----RGVVYGGMIRELLLSFKRA-
MtAGO12c  513 --------------------------------------------------------------------------------------------------------------
AtAGO5    698 VLNDAIRRNIPLI--TDRPTIIMGADVTHPQPGED-SSPSIAAVVA----SMDWPEINKYRG-LVSAQAHREEIIQDLYKLVQDPQ----RGLVHSGLIREHFIAFRRA-
AtAGO7    709 ELYNSIPSHIPRLLRPDEPVIFMGADVTHPHPFDD-CSPSVAAVVG----SINWPEANRYVS-RMRSQTHRQEIIQDLD-----------------LMVKELLDDFYKA-
MtAGO7    724 ALYNSLPSQLPRLFNIDEPVMFMGADVTHPHPLDD-SSPSVAAVVG----SMNWPTANKYIS-RIRSQTHRQEIIADLG-----------------AMVGELLEDFYQE-
AtAGO2    726 ELMDTFS----FF-KKEDEVMFIGADVNHPAARDK-MSPSIVAVVG----TLNWPEANRYAA-RVIAQPHRKEEIQGFG-----------------DACLELVKAHVQA-
AtAGO3    834 ELVDNIFS---FF-KKEDKVMFIGADVNHPAAHDN-MSPSIVAVVG----TLNWPEANRYAA-RVKAQSHRKEEIQGFG-----------------ETCWELIEAHSQA-
MtAGO2a   627 ELINRLP----HF-EDESHVMFIGADVNHPGSRDT-NSPSIVAVVA----TTNWPAANRYAA-RVCAQEHCTEKILNFG-----------------EICLDLVRHYEKL-
MtAGO2b   598 ELNNRLP----YF-EGEEHVMFIGADVNHPGSRDN-KSPSIVAVVA----TINWPAANRYAA-RVCPQFNRSEKILNFG-----------------EICVELVSCYWQK-
AtAGO6    600 LLGIEYSYNIPLI--NKIPTLILGMDVSHGPPGRA-DVPSVAAVVG----SKCWPLISRYRA-AVRTQSPRLEMIDSLFQPIENTE------KGDNGIMNELFVEFYRTS
MtAGO6    642 LLAIEHSGHLPLI--KDTPTMILGMDVSHGSPGRS-DIPSIAAVVG----SRCWPLISRYRA-SVRSQSPKVEMIDSLFKLVDKMNDDGILEKKDDGIIRELLLDFYSSS
MtAGO11   602 FLLTEFKHSIPLF--SKIPTLVIGMDVSHGSQGQS-EALSIAAVVS----SRCWPQISRYKA-VVRTQSSKVEIVQSLFKPVSDT--------KDDGIISELLKDFQTT-
AtAGO8    681 VLDMELSGTMPLV--MRVPTIIIGMDVSHGSPGQSDHIPSIAAVVS----SREWPLISKYRA-CVRTQSPKVEMIDSLFKPVSDK--------DDQGIMRELLLDFHSS-
AtAGO9    609 LLAMERSPAMPKV--TQVPTIIVGMDVSHGSPGQS-DIPSIAAVVS----SRQWPLISKYKA-CVRTQSRKMEMIDNLFKPVNG---------KDEGMFRELLLDFYYSS
AtAGO4    637 MLSVERTPAFTVI--SKVPTIILGMDVSHGSPGQS-DVPSIAAVVS----SREWPLISKYRA-SVRTQPSKAEMIESLVKKN-GT--------EDDGIIKELLVDFYTSS
MtAGO4a   536 LLGVESSPSLPIV--SKAPTLILGMDVSHGSPGQT-DIPSIAAVVS----SRQWPLISKYRA-CVRTQSAKVEMIDNLFKKVSDT--------EDEGIMRELLLDFYTSS
MtAGO4b   653 LLGVEHSPSIPIV--SKAPTLILGMDVSHGSPGQT-EIPSIAAVVS----SRQWPLISKYRA-CVRTQGAKVEMIDNLFKPVSDT--------EDEGIIRELLIDFYNSS
MtAGO4c   627 WLGVEHSRSIPIV--SKVPTLILGMDVSHGSPGQP-DIPSIAAVVS----SRKWPLISKYRA-CVRTQGSKVEMIDNLFKPVSDK--------EDEGIIRELLLDFFHSS
TtAGO     464 ALSG------AY-----PAELAVGFDAGGRESF--R-FGGAACAVGGDGGHLLWTL-PEAQA----GERIPQEVVWD--------------------LLEETLWAFRRK-
PfAGO     544 VLDYRFN-----------YDYIIGIDVAPMKRSEG-YIGGSAVMFD----SQGY--IRKI-V-PIKIGEQRGESV-DM-----------------NEFFKEMVDKFKEFN
AaAGO     487 KL--------KEI--EGKVDAFVGIDISRITRDGK-TVNAVAFTKI----FNSKGELVRYYLTSYPAFGEK----------------------LTEKAIGDVFSLLEK-L
HsPIWI    731 GVDI------PL-----KQLMVIGMDVYHDPSR--G-MRSVVGFVA----SINLTL-TKWYS-RVVFQMPHQEIVDSLK-----------------LCLVGSLKKFYEV-
HsAGO1    575 ILVPH---QRSAV--FQQPVIFLGADVTHPPAGDG-KKPSITAVVG----SMDA-HPSRYCA-TVRVQRPRQEIIEDLS-----------------YMVRELLIQFYKS-
HsAGO2    577 ILLPQ---GRPPV--FQQPVIFLGADVTHPPAGDG-KKPSIAAVVG----SMDA-HPNRYCA-TVRVQQHRQEIIQDLA-----------------AMVRELLIQFYKS-

AtAGO1        TGHKPLRIIFYRDG-VSEGQFYQVLLYELDAIRKACASLEAGYQPPVTFVVVQKRHHTRLFAQNHNDRHS----VDRSGNILPGTVVDSKICHPTEFDFY-LCS-HAGIQ
MtAGO1        TGQKPQRIIFYRDG-VSEGQFYQVLLFELDAIRKACASLEPNYQPPVTFVVVQKRHHTRLFASNHQDKSS----VDRSGNILPGTVVDSKICHPTEFDFY-LCS-HAGIQ
AtAGO10       TGQKPLRIIFYRDG-VSEGQFYQVLLYELDAIRKACASLEPNYQPPVTFIVVQKRHHTRLFANNHRDKNS----TDRSGNILPGTVVDTKICHPTEFDFY-LCS-HAGIQ
MtAGO12a      TGKKPCRILFYRDG-VSEGQFYQVLLYELDAIRKACASLEPGYQPPVTFVVVQKRHHTRLFSDNHNDRNS----MDRSGNILPGTVVDTKICHPTEFDFY-LCS-HAGVQ
MtAGO12b      TGQKPLRIIFYRDG-VSEGQFYQVLLYELDAIRKACASLEPSYQPPVTFVVVQKRNHTRLFPNNHDDRNS----TDTSGNILPGTVVDSKICHPTEFDFY-LCS-HAGIQ
MtAGO12c      --------------------------------------------------------------------------------------------------------------
AtAGO5        TGQIPQRIIFYRDG-VSEGQFSQVLLHEMTAIRKACNSLQENYVPRVTFVIVQKRHHTRLFPEQHGNRDM----TDKSGNIQPGTVVDTKICHPNEFDFY-LNS-HAGIQ
AtAGO7        VKKLPNRIIFFRDG-VSETQFKKVLQEELQSIKTACSKF-QDYNPSITFAVVQKRHHTRLFRCDPD-----------HENIPPGTVVDTVITHPKEFDFY-LCS-HLGVK
MtAGO7        VEKLPNRIIFFRDG-VSETQFYKVLQEELQSIKQACSSRFHGYKPFITFVVVQKRHHTRLFPADTDQSSMHNNFHFQYENIPPGTVVDSVITHPKEFDFY-LCS-HWGVK
AtAGO2        TGKRPNKIVIFRDG-VSDAQFDMVLNVELLDVKLTFEK--NGYNPKITVIVAQKRHQTRFFPATNND-------GSDKGNVPSGTVVDTKVIHPYEYDFY-LCS-HHGGI
AtAGO3        PEKRPNKIVIFRDG-VSDGQFDMVLNVELQNVKDVFAK--VGYNPQITVIVAQKRHQTRFFPATTSKD------GRAKGNVPSGTVVDTTIIHPFEYDFY-LCS-QHGAI
MtAGO2a       NKVRPQKIVIFRDG-VSESQFHMVLGEELKDLKTVFQH--SNYFPTITLIVAQKRHQTRLFPAGVRE-------GAPSGNVFPGTVVDTKVVHPFEFDFY-LCS-HYGSL
MtAGO2b       NGVRPEKIVVFRDG-VSEFQFDMVLNEELLDLKRAFQR--LNYFPTITLIVAQKRHQTRFFPDSWRD-------GSSSGNILPGTVVDTKVTHPFEFDFY-LCS-YYGSL
AtAGO6        RARKPKQIIIFRDG-VSESQFEQVLKIEVDQIIKAYQRLGESDVPKFTVIVAQKNHHTKLFQAKGP------------ENVPAGTVVDTKIVHPTNYDFY-MCA-HAGKI
MtAGO6        GNRRPTQIILFRDG-VGESQFQHVLDIELNQIIKAYKHID-GDVPKFTVIVAQKNHHTKLFQANALE-----------KNVPPGTVVDTNIVHPRNYDFY-MCA-HAGMI
MtAGO11       SGVKPQQIIIFRDG-VSESQFNQVLNIELNEIIKACKCYDESWCPKFTLIVAQKNHHTRFFKANSPQ-----------ENVSPGTVIDNTICHPKDNDFY-MCA-HAGRI
AtAGO8        SGKKPNHIIIFRDG-VSESQFNQVLNIELDQMM-------------------QINHHTKFFQTESP------------NNVLPGTIIDSNICHQHNNDFY-LCA-HAGKI
AtAGO9        ENRKPEHIIIFRDG-VSESQFNQVLNIELDQMMQACKFLDDTWHPKFTVIVAQKNHHTKFFQSRGP------------DNVPPGTIIDSQICHPRNFDFY-LCA-HAGMI
AtAGO4        NKRKPEHIIIFRDG-VSESQFNQVLNIELDQIIEACKLLDANWNPKFLLLVAQKNHHTKFFQPTSP------------ENVPPGTIIDNKICHPKNNDFY-LCA-HAGMI
MtAGO4a       KNRKPDNIIIFRDG-VSESQFNQVLNIELDQIIEACKFLDENWTPKFVVIVAQKNHHTRFFQPNSP------------DNVPPGTVIDNKICHPRNYDFY-LCA-HAGMI
MtAGO4b       GNRKPDNIIIFRDG-VSESQFNQVLNIELSQIIEACKFLDEKWNPKFLVIVAQKNHHTKFFQPGSP------------DNVPPGTVVDNKICHPRNYDFY-MCA-HAGMI
MtAGO4c       EERRPENIIIFRDG-VSESQFNEVLNVELSQIIEACKFLDENWNPKFMVIVAQKNHHTKFFQPRSP------------DNVPPGTVVDSKICHPRNYDFY-MCA-HAGMI
TtAGO         AGRLPSRVLLLRDGRVPQDEFALALEALAREG------------IAYDLVSVRKSGGGRVYPV-------------------QGRLADGLYVPLEDKTFLLLTV-HRDFR
PfAGO         IKLDNKKILLLRDG-RITNNEEEGLK-----------YISEMFDIEVVTMDVIKNHPVRAFANMKMYF-------------------------NLGGAIY-LIP-HKLKQ
AaAGO         GFKKGSKIVVHRDG-RLYR-------DEVA----AFKKYGELYGYSLELLEIIKRNNPRFFSNEKFIKGY--------------------FYKLSEDSVIL-ATYNQVYE
HsPIWI        NHCLPEKIVVYRDG-VSDGQLKTVANYEIPQLQKCFEA-FENYQPKMVVFVVQKKISTNLYLAAPQNF----------VTPTPGTVVDHTITSCEWVDFY-LLA-HHVRQ
HsAGO1        TRFKPTRIIFYRDG-VPEGQLPQILHYELLAIRDACIKLEKDYQPGITYIVVQKRHHTRLFCADKNER------IGKSGNIPAGTTVDTNITHPFEFDFY-LCS-HAGIQ
HsAGO2        TRFKPTRIIFYRDG-VSEGQFQQVLHHELLAIREACIKLEKDYQPGITFIVVQKRHHTRLFCTDKNER------VGKSGNIPAGTTVDTKITHPTEFDFY-LCS-HAGIQ

AtAGO1        GTSRPAHYHVLW---------DENNFTA-DGLQSLTNNLCYTYARCTRSV-SIVPPAYYAHLAAFRARFY-M 998
MtAGO1        GTSRPAHYHVLW---------DENNFSA-DGLQSLTNNLCYTYARCTRSV-SIVPPAYYAHLAAFRARFY-M 992
AtAGO10       GTSRPAHYHVLW---------DENNFTA-DGIQSLTNNLCYTYARCTRSV-SIVPPAYYAHLAAFRARFY-L 945
MtAGO12a      GTSKPAHYHVIW---------DDNKFSA-DEIQSLTNNLCYTYARCTRSV-SLVPPAYYAHLAAYRARFY-M 839
MtAGO12b      GTSRPAHYHVLW---------DENNFSP-DEIQSLTNNLCYTYARCTRSV-SVVPPAYYAHLAAYRARCY-M 557
MtAGO12c      ------------------------------------------------------------------------ 513
AtAGO5        GTSRPAHYHVLL---------DENGFTA-DQLQMLTNNLCYTYARCTKSV-SIVPPAYYAHLAAFRARYY-M 957
AtAGO7        GTSRPTHYHILW---------DENEFTS-DELQRLVYNLCYTFVRCTKPI-SIVPPAYYAHLAAYRGRLY-I 949
MtAGO7        GTSRPTHYHVLL---------DENKFTS-DELQKLVYNLCFTFVRCTKPI-SLVPPAYYAHLAAYRGRLY-L 976
AtAGO2        GTSKPTHYYTLW---------DELGFTS-DQVQKLIFEMCFTFTRCTKPV-SLVPPVYYADMVAFRGRMY-H 964
AtAGO3        GTSKPTHYYVLS---------DEIGFNS-NQIQKLIFDLCFTFTRCTKPV-ALVPPVSYADKAASRGRVY-Y 1074
MtAGO2a       GTSKPTHYHVLW---------DEHRFTS-DNLQKLIYDMCFTFARCTKPV-SLVPPVYYADLAAYRGRLY-Y 865
MtAGO2b       GTSKPTHYHVLW---------DEHKFTS-DELQKLIYEMCFTFARCTKPV-SLVPPVYYADLAAYRGRLY-H 836
AtAGO6        GTSRPAHYHVLL---------DEIGFSP-DDLQNLIHSLSYVNQRSTTAT-SIVAPVRYAHLAAAQVAQF-T 850
MtAGO6        GTSRPVHYHVLL---------DEIGFSS-DGLQNLINSLSYVNQRSTAAT-SIVAPIYYAHHAAAQMRKF-M 898
MtAGO11       GTSRPTHYHVLY---------DEIGFSA-DNLQEFVHSLCYVHQRSTNAI-SIVAPIYYADLAAAQIAQF-I 850
AtAGO8        GTTRPTHYHVLY---------DEIGFDT-DQLQELVHSLSYVYQRSTTAI-SLVAPICYAHLAAAQMATA-M 810
AtAGO9        GTTRPTHYHVLY---------DEIGFAT-DDLQELVHSLSYVYQRSTTAI-SVVAPVCYAHLAAAQMGTV-M 856
AtAGO4        GTTRPTHYHVLY---------DEIGFSA-DELQELVHSLSYVYQRSTSAI-SVVAPICYAHLAAAQLGTF-M 884
MtAGO4a       GTSRPTHYHVLL---------DEIGFSP-DELQELGHSLSYVYQRSTTAI-SVVAPICYAHLAATQLGQF-M 784
MtAGO4b       GTSRPTHYHVLL---------DEIGFSP-DDLQELVHSLSYVYQRSTTAI-SVVAPICYAHLAASQVGQF-M 901
MtAGO4c       GTSRPTHYHVLL---------DEIGFSP-DDLQELVHSLSYVYQRSTTAI-SVVAPICYAHLAASQVGQF-M 875
TtAGO         GTPRPLKL---V---------HEAGDTPLEALAHQIFHLTRLYPASGFAFPRLPAPLHLADRLVKEVGRLGI 671
PfAGO         AKGTPIPIKLAKKRIIKNGKVEKQSITR-QDVLDIFILTRLNYGSISADM-RLPAPVHYAHKFANAIRNE-W 755
AaAGO         GTHQPIKVRKVY---------GELPVE---VLCSQILSLTLMNYSSFQPI-KLPATVHYSDKITKLMLRG-I 693
HsPIWI        GCGIPTHYVCVL---------NTANLSP-DHMQRLTFKLCHMYWNWPGTI-RVPAPCKYAHKLAFLSGHI-L 958
HsAGO1        GTSRPSHYYVLW---------DDNRFTA-DELQILTYQLCHTYVRCTRSV-SIPAPAYYARLVAFRARYH-L 815
HsAGO2        GTSRPSHYHVLW---------DDNRFSS-DELQILTYQLCHTYVRCTRSV-SIPAPAYYAHLVAFRARYH-L 817
```
